# Increased Post-Hypoxic Oxidative Stress and Activation of the PERK Branch of the UPR in *Trap1*-Deficient *Drosophila melanogaster* Is Abrogated by Metformin

**DOI:** 10.3390/ijms222111586

**Published:** 2021-10-28

**Authors:** Alma Kokott-Vuong, Jennifer Jung, Aaron T. Fehr, Nele Kirschfink, Rozina Noristani, Aaron Voigt, Arno Reich, Jörg B. Schulz, Michael Huber, Pardes Habib

**Affiliations:** 1 Department of Neurology, Medical Faculty, RWTH Aachen University, 52074 Aachen, Germany; alma.kokott@rwth-aachen.de (A.K.-V.); jennifer.jung@rwth-aachen.de (J.J.); aaron.fehr@rwth-aachen.de (A.T.F.); nele.kirschfink@rwth-aachen.de (N.K.); rozinanoristani@gmail.com (R.N.); avoigt@ukaachen.de (A.V.); areich@ukaachen.de (A.R.); jschulz@ukaachen.de (J.B.S.); 2 JARA-BRAIN Institute Molecular Neuroscience and Neuroimaging, Forschungszentrum Jülich GmbH and RWTH Aachen University, 52074 Aachen, Germany; 3 Institute of Biochemistry and Molecular Immunology, Medical Faculty, RWTH Aachen University, 52074 Aachen, Germany; mhuber@ukaachen.de

**Keywords:** Hsp90 family, Hsp75, mitochondrial chaperone, ER-stress, ROS, stroke, hypoxia, ischemia

## Abstract

Hypoxia is known to impair mitochondrial and endoplasmic reticulum (ER) homeostasis. Post-hypoxic perturbations of the ER proteostasis result in the accumulation of misfolded/unfolded proteins leading to the activation of the Unfolded Protein Response (UPR). Mitochondrial chaperone TNF receptor-associated protein 1 (TRAP1) is reported to preserve mitochondrial membrane potential and to impede reactive oxygen species (ROS) production thereby protecting cells from ER stress as well as oxidative stress. The first-line antidiabetic drug Metformin has been attributed a neuroprotective role after hypoxia. Interestingly, Metformin has been reported to rescue mitochondrial deficits in fibroblasts derived from a patient carrying a homozygous TRAP1 loss-of-function mutation. We sought to investigate a putative link between Metformin, TRAP1, and the UPR after hypoxia. We assessed post-hypoxic/reperfusion longevity, mortality, negative geotaxis, ROS production, metabolic activity, gene expression of antioxidant proteins, and activation of the UPR in *Trap1*-deficient flies. Following hypoxia, *Trap1* deficiency caused higher mortality and greater impairments in negative geotaxis compared to controls. Similarly, post-hypoxic production of ROS and UPR activation was significantly higher in *Trap1*-deficient compared to control flies. Metformin counteracted the deleterious effects of hypoxia in *Trap1*-deficient flies but had no protective effect in wild-type flies. We provide evidence that TRAP1 is crucially involved in the post-hypoxic regulation of mitochondrial/ER stress and the activation of the UPR. Metformin appears to rescue *Trap1*-deficiency after hypoxia mitigating ROS production and downregulating the pro-apoptotic PERK (protein kinase R-like ER kinase) arm of the UPR.

## 1. Introduction

Hypoxia is a key underlying condition of various devastating diseases including pulmonary hypertension, ischemic heart failure as well as global and focal cerebral ischemia [1,2,3]. Acute ischemic stroke is one of the main causes of death and disability in adults worldwide [3,4,5]. Here, occlusion of a cerebral artery results in a rapid deprivation of oxygen and nutrients leading to neuronal cell death [3,5]. Excitotoxicity, oxidative stress, endoplasmic reticulum (ER) stress, and mitochondrial impairment are the most frequently observed mechanisms after hypoxia/ischemia [6]. Post-hypoxic oxidative stress and excitotoxicity, caused by a substantial release of glutamate, lead to an accumulation of Ca^2+^ in mitochondria [6]. Elevated Ca^2+^ levels open the mitochondrial permeability transition pore (MPTP) releasing cytochrome C (CytC) into the cytosol and inducing a breakdown of the membrane potential accompanied by an osmotic swelling of mitochondria [6,7]. Cytosolic CytC forms a complex with the initiator caspase 9, which in turn activates the effector caspases 3 or 7 resulting in apoptosis [7,8,9,10,11]

In addition, hypoxia leads to a depletion of ATP and a generation of reactive oxygen species (ROS) due to reduced oxidative phosphorylation through ATP-Synthase (Complex IV) and the impaired mitochondrial membrane polarization [8,9,10,11,12,13]. ROS has been reported to cause ER-stress. The latter is defined as the result of an imbalance between a load of unfolded proteins that enter the ER and its folding capacity [14]. As the ER is also the intracellular Ca^2+^ store, a vicious cycle condition arises due to the released calcium during ER-stress and its influence on mitochondrial ROS production [12,15].

Following ER stress, a highly conserved intracellular mechanism termed the Unfolded Protein Response (UPR) is initiated to resolve ER stress (adaptive UPR) [16]. However, sustained unresolved ER stress might result in cell death (terminal UPR) [17]. The UPR consists of three branches, each regulated by ER transmembrane sensors: IRE1α (inositol-requiring enzyme 1α), PERK (protein kinase R-like ER kinase), and ATF6 (activating transcription factor 6) [14,18,19]. IRE1α cleaves the mRNA coding for the X-box binding protein (XBP1), which encodes the transcriptional activator spliced XBP1 (XBP1s). XBP1s regulate the expression of ER chaperones and proteins promoting ER-associated degradation (ERAD) of misfolded proteins [14,18,20]. Activation of the PERK arm leads to reduced numbers of newly synthesized proteins but also favors the induction of the transcription factor ATF4. The latter is reported to be crucially involved in the induction of apoptosis upon sustained UPR [14,17,18,21]. ATF6 is known to regulate the expression of genes encoding ERAD components and ER chaperones [14,17,18,22].

TNF receptor-associated protein 1 (TRAP1), also known as Heat-shock protein 75 (HSP75), is a mitochondrial chaperone that is attributed to protecting cells against ER stress [23,24]. Takemoto et al. suggested that the HSP90 family member TRAP1 might regulate the UPR in the ER and that its downregulation in SH-SY5Y neuroblastoma cells enhances Caspase-4, which has been associated with ER stress-induced cell death [25,26]. Increased levels of TRAP1 were found in tumor cells compared to healthy tissue. Here, lower expression abundance levels of TRAP1 positively correlated with elevated mitochondrial impairment and cell death [27]. Silencing TRAP1 in-vitro has been described to enhance the release of CytC causing Granzyme B-induced death [28]. An upregulation of TRAP1 was reported after various acute stress conditions including glucose deprivation (GD), oxidative injury, ultraviolet A irradiations, and cerebral ischemia [29,30,31,32]. Of note, overexpression of TRAP1 appears to attenuate ROS production [33]. However, the exact mechanisms of TRAP1-mediated anti-apoptotic effects and the impact of TRAP1 on the UPR and ER stress under hypoxia have not been investigated yet.

Recently Fitzgerald and colleagues demonstrated that a homozygous TRAP1 loss-of-function mutation in fibroblasts derived from a patient-led mitochondrial dysfunction and was rescued by the first-line antidiabetic drug Metformin [34]. Metformin is widely known to efficiently and safely lower blood glucose levels [35,36]. It is also associated with renoprotective properties as well as protection against cancer and beneficial effects on polycystic ovary syndrome [37]. Although Metformin has previously been described as neuroprotective after experimental and clinical ischemic stroke, its mechanism of action remains elusive [38,39]. Previous studies highlighted the antioxidant role of Metformin. Indeed, Metformin decreased intracellular ROS production in aortic endothelial cells but also increased the antioxidant activities of Superoxide dismutase (SOD) and Catalase [40,41]. Therefore, we sought to investigate a connection between Metformin, TRAP1, and the post-hypoxic activation of the UPR.

We assessed the post-hypoxic longevity, mortality, negative geotaxis, ROS production, metabolic activity, gene expression of *Sod, Catalase,* and *Hsp70*, and the UPR activation in *Trap1*-deficient *Drosophila melanogaster (D. melanogaster*) and corresponding wild-type controls (Canton-S). *D. melanogaster* as a model species has many advantages such as a short life cycle, large numbers of progeny, limited need for resources, and a remarkably high degree of conservation compared to the human genome [42,43,44]. Our established hypoxia system for *D. melanogaster* with the ability to monitor and regulate parameters such as oxygen levels, temperature, humidity, and atmospheric pressure enabled us to induce reproducible and valid hypoxia during each experiment [45]. 

In this study, we demonstrate that *Trap1* deficiency leads to higher mortality rates, a significantly lower lifespan, and a reduced negative geotaxis in flies after hypoxia. On the molecular level, *Trap1*-deficient flies display an elevated ROS production and higher metabolic activity as well as an upregulation of UPR markers and *Sod, Catalase,* and *Hsp70* mRNAs compared to Canton-S. Metformin counteracts these post-hypoxic effects of *Trap1* deficiency by leading to reduced mortality rates through lower ROS production levels and downregulation of the pro-apoptotic PERK branch in *Trap1*-deficient compared to wildtype Canton-S flies after hypoxia.

We provide evidence that TRAP1 plays an important role in the post-hypoxic regulation of mitochondrial/ER stress and the activation of the UPR. Metformin appears to rescue the effects of *Trap1* deficiency after hypoxia through a reduction of ROS levels and a downregulation of the pro-apoptotic PERK branch of the UPR.

## 2. Results

### 2.1. Trap1 Deficiency Leads to Increased Mortality Rates after Severe Hypoxia (<0.3% O_2_) in D. melanogaster

**Figure 1:**
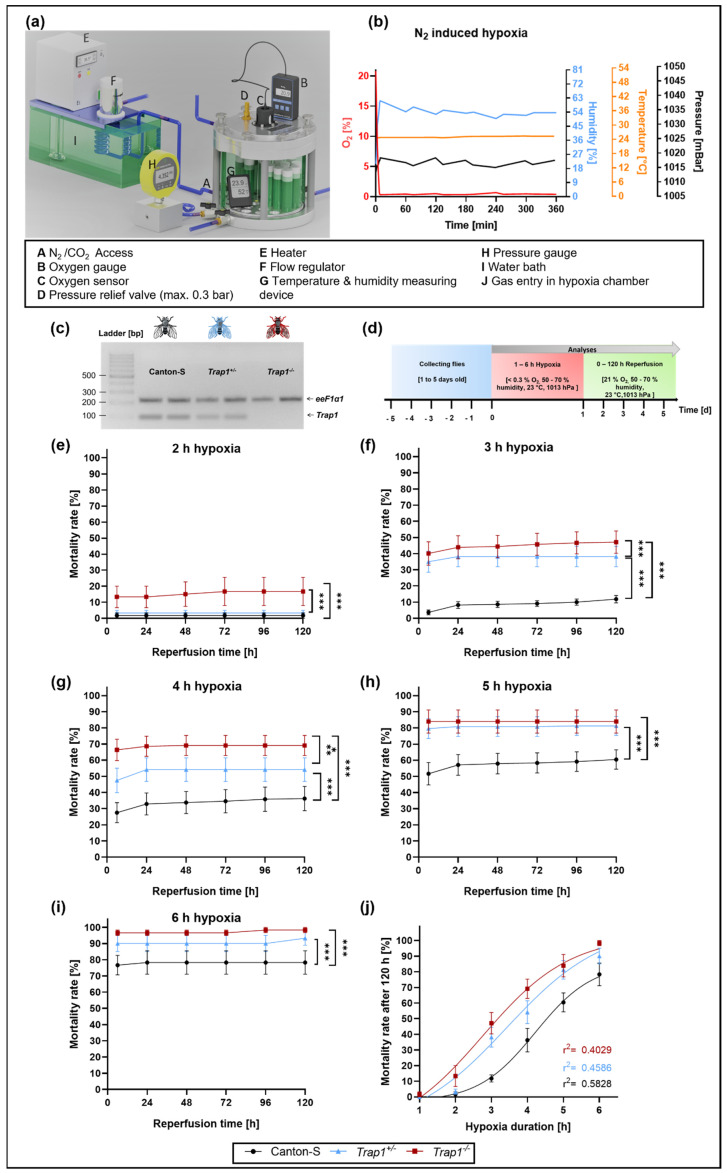
Mortality rates of Canton-S, *Trap1*^+/−^ and *Trap1*^−/−^ flies subjected to different durations of monitored N_2_-induced hypoxia (<0.3% O_2_). (**a**) Hypoxia chamber used for the experiments. Illustration of hypoxia chamber and environmental conditions modified from Habib et al. 2021 (**b**) Hypoxia conditions at 23 °C with controlled oxygen levels, pressure, and humidity. (**c**) Genotypes of flies assessed by PCR. (**d**) Schematic illustration of the hypoxia-reperfusion protocol. (**e**–**i**) Death rates of Canton-S, *Trap1*^+/−^ and *Trap1*^−/−^ after 1 to 6 h of severe hypoxia (<0.3% O_2_) followed by a reperfusion period of 120 h. (**j**) Nonlinear regression of the hypoxia duration-dependent death rates of Canton-S, *Trap1*^+/−^ and *Trap1*^−/−^ *D. melanogaster*. Data are shown as means ± SEM of 4 independent experiments, including 3 technical replicates per genotype and experiment (total of 240 male flies for each genotype and condition). Repeated measurements one-way ANOVA followed by Tukey post-hoc. * *p* < 0.05., ** *p* < 0.01, *** *p* < 0.001.

To assess the impact of TRAP1 on the mortality rates of *D. melanogaster* after severe hypoxia, we subjected wild-type flies (Canton-S) as well as *Trap1*-deficient flies (*Trap1* heterozygous (*Trap1*^+/−^) and *Trap1* homozygous (*Trap1*^−/−^), respectively) to severe hypoxia in a self-designed hypoxia chamber under controlled conditions followed by a reperfusion period of 120 h (Figure 1a–d). With the increasing duration of hypoxia, we observed increasing mortality rates in all genotypes. *Trap1*-deficient flies revealed significantly higher mortality rates after all hypoxia durations compared to Canton-S flies (*p* < 0.0001). Thereby, homozygous *Trap1*-deficient flies consistently showed the highest level of mortality (Figure 1c–i). While a hypoxia duration of 3 h induced a mortality rate of 47.12% in *Trap*^−/−^, only 38.18% *Trap1*^+/−^ flies died after 120 h of reperfusion and 11.82% of Canton-S died in the same period (Figure 1f). Nonlinear regression analysis of the mortality rates after 120 h reperfusion following 1–6 h hypoxia displayed enhanced death rates in *Trap1*-deficient flies (Figure 1j). In conclusion, *Trap1* deficiency increases severe hypoxia-induced mortality rates compared to the wildtype Canton-S flies.

### 2.2. Trap1 Deficiency Impairs Lifespan, Activity Rates, and Negative Geotaxis after Hypoxia in D. melanogaster

To monitor the activity and mortality of *D. melanogaster* after hypoxia we subjected Canton-S and *Trap1*-deficient flies to 3 h of hypoxia and afterward assessed their activity in a *Drosophila* Activity Monitoring (DAM) system for 5 days.

**Figure 2:**
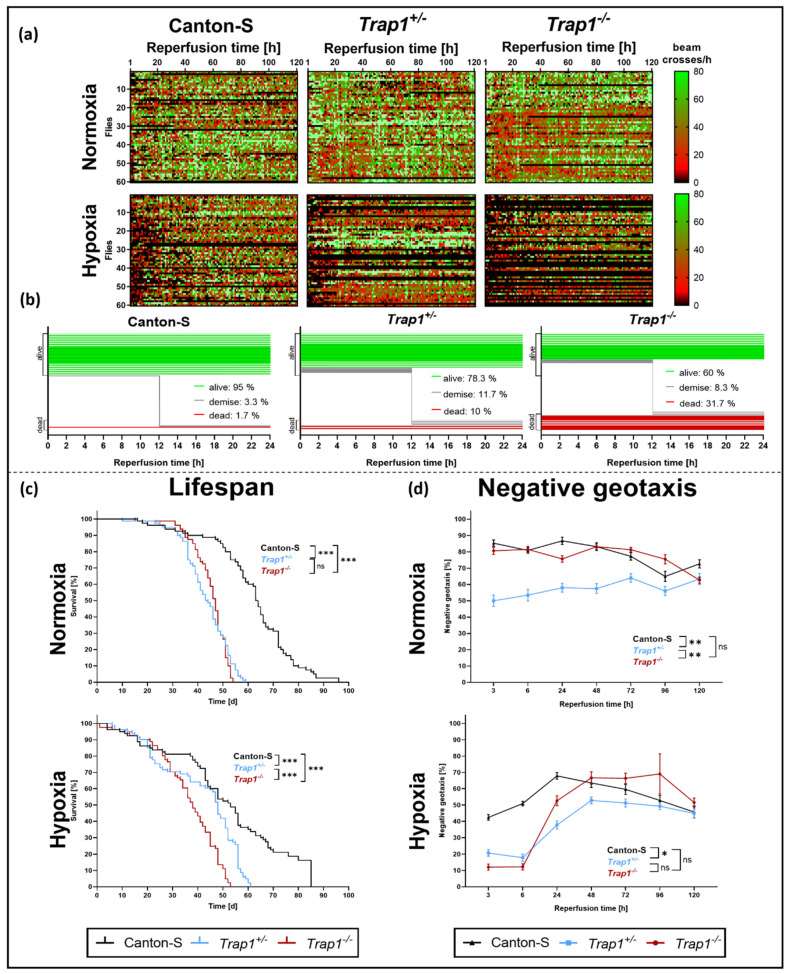
Activity, lifespan, and negative geotaxis of Canton-S, *Trap1*^+/−^ and *Trap1*^−/−^ *D. melanogaster* after 3 h of severe hypoxia followed by 120 h of reperfusion. (**a**) Heatmaps displaying the activity of Canton-S, *Trap1*^+/−^ and *Trap1*^−/−^ after 3 h of severe hypoxia (<0.3% O_2_) or normoxia (21% O_2_) followed by 120 h of reperfusion. Each cell shown is the absolute value of beam crosses per hour per fly, represented by color. Black: no activity, red: moderate activity, green: high activity. (**b**) Quantification of survival, instant death, and reperfusion-dependent death of Canton-S, *Trap1*^+/−^ and *Trap1*^−/−^ *D. melanogaster* in the first 24 h after 3 h of severe hypoxia (<0.3% O_2_). (**c**) Kaplan-Mayer-curves of Canton-S, *Trap1*^+/−^ and *Trap1*^−/−^ flies subjected to 3 h of hypoxia or normoxia. The statistical significance was determined using the chi-squared-based log-rank, Mantel-Cox test. * *p* < 0.05, ** *p* < 0.01, *** *p* < 0.001 (**d**) Mortality adjusted negative geotaxis of Canton-S, *Trap1*^+/−^ and *Trap1*^−/−^ *D. melanogaster* subjected to 3 h of hypoxia or normoxia followed by 120 h of reperfusion. The statistical significance was determined using repeated measurements one-way ANOVA followed by Tukey’s post-hoc test (multiple comparisons). Data represent means ± SEM from 3 (**d**) to 4 (**a**–**c**) independent experiments including 60 male flies per experiment and genotype.

Hypoxia led to reduced activity and higher mortality rates compared to normoxia. The activity rates in *Trap1*-deficient flies were markedly reduced compared to Canton-S flies (Figure 2a). Moreover, *Trap1*-deficient flies displayed a higher instant death rate after hypoxia of 10.0% in *Trap1*^+/−^ and 31.7% in *Trap1*^−/−^ compared to 1.7% in Canton-S flies as well as a higher demise rate of 8.3% and 11.7% compared to 3.3% in Canton-S during the first 24 h of reperfusion (Figure 2b).

Investigating the lifespan we used 1-day old flies of all three genotypes and performed 3 h hypoxia. Noteworthy, in contrast to our mortality assessment younger flies were taken for the lifespan assessment, which might explain the lower mortality rate in the first five days after hypoxia.

Under normoxia, *Trap1*-deficient flies had a significantly reduced lifespan compared to Canton-S (*p* < 0.0001). Indeed, the median overall survival was reached after 63 days for Canton-S while *Trap1^+/−^* flies already reached it after 43 days and *Trap1*^−/−^ flies after 47 days. Hypoxia-induced increased death rates in all genotypes at the beginning of the observation period. Here the median overall survival was reached after 53 days in Canton-S, 48 days in *Trap1^+/^*^−^ and 38 days in *Trap1*^−/−^. However, the maximal life expectancy was not affected by hypoxia (Figure 2c). 

Assessing the negative geotaxis, *Trap1*^+/−^ revealed a significantly reduced climbing ability under normoxic conditions compared to Canton-S (*p* = 0.0043) and *Trap1*^−/−^ (*p* = 0.0058). After hypoxia, we observed an initial reduction of negative geotaxis in *Trap1*-deficient flies compared to Canton-S (*p* < 0.000001) during the first 24 h of reperfusion, which recovered after 48 h (Figure 2d). 

In conclusion, *Trap1* deficiency leads to a significantly lower lifespan in both normoxia and hypoxia and negatively affects the activity and negative geotaxis.

### 2.3. Hypoxia-Dependent Metabolic Activity and ROS Production Are Increased in Trap1-Deficient Flies

**Figure 3:**
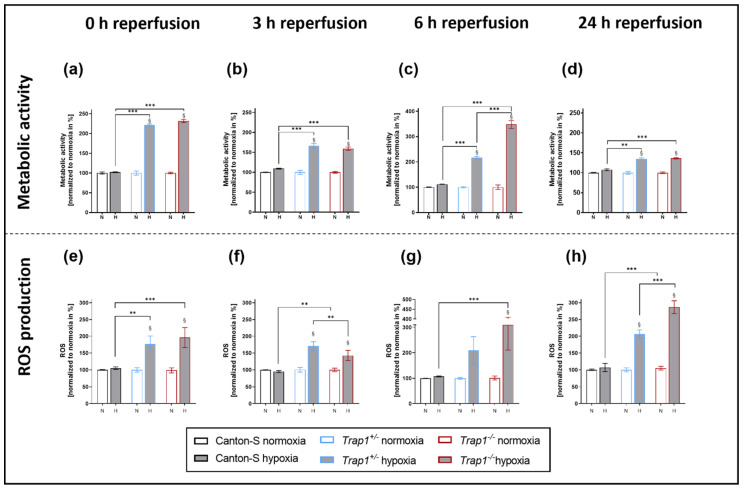
Metabolic activity and ROS production of Canton-S, *Trap1*^+/−^ and *Trap1*^−/−^ flies during a reperfusion period of 24 h. (**a**–**d**) Metabolic activity in the supernatant in fly head lysates after 3 h of hypoxia. ROS production in the fly head lysates is shown in (**e**–**h**)**.** The graphs present the means ± SEM of 4 independent experiments. Two-way ANOVA followed by Tukey post-hoc. ** *p* < 0.01, *** *p* < 0.001. ^§^ *p* < 0.05 indicates significance compared to corresponding normoxia.

To assess the metabolic activity, we performed a CellTiter-Blue^®^ Cell Viability Assay after 3 h of hypoxia and different reperfusion times. While no difference between the genotypes was observed under normoxic conditions, the metabolic activity of both *Trap1*^+/−^ and *Trap1*^−/−^ flies was significantly enhanced after hypoxia compared to the corresponding normoxia control at every reperfusion time point (*p* < 0.0001), peaking at 6 h of reperfusion in the *Trap*^−/−^ flies. Meanwhile, Canton-S displayed no difference in metabolic activity between hypoxia and normoxia (Figure 3a–d).

Investigating the oxidative stress, we analyzed the reactive oxygen species (ROS) levels in fly heads at different reperfusion times after 3 h of hypoxia. While the ROS levels of Canton-S did not differ between hypoxia and normoxia, *Trap1*-deficient flies demonstrated significantly higher ROS levels after hypoxia compared to normoxia during the whole reperfusion period (*p* < 0.0001). We observed a significant increase of ROS production after hypoxia in *Trap1*^−/−^ flies after 0 h (*p* < 0.0001), 3 h (*p* < 0.0001), 6 h (*p* < 0.0001), and 24 h (*p* < 0.0001), and in *Trap1*^+/−^ flies after 0 h (*p* = 0.0026), 3 h (*p* < 0.0001), and 24 h (*p* < 0.0001) of reperfusion, whereas Canton-S presented no difference after hypoxia compared to normoxia (Figure 3e–h).

In conclusion, *Trap1* deficiency leads to significantly enhanced metabolic activity and ROS production after hypoxia at all reperfusion timepoints which might explain the higher mortality in *Trap1*-deficient flies.

### 2.4. Metformin Rescues Hypoxia-Dependent Mortality in Trap1-Deficient Flies and Impairs the Expression Pattern of Trap1 mRNA

**Figure 4:**
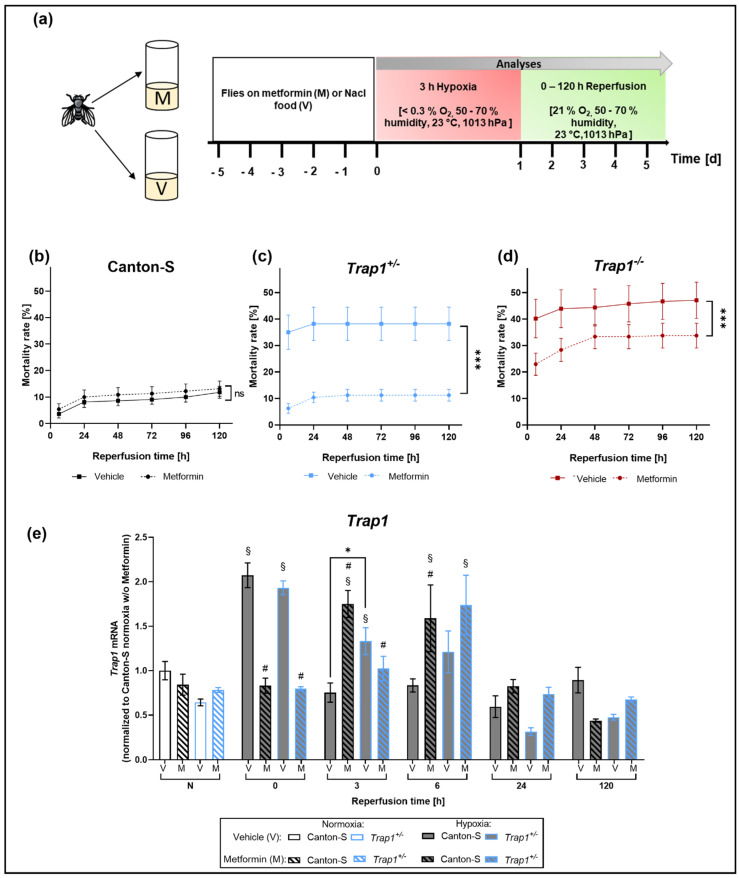
Hypoxia-dependent mortality and mRNA levels of *Trap1* in Canton-S and *Trap1*-deficient flies after Metformin treatment. (**a**) Schematic illustration of hypoxia protocol after Metformin treatment. (**b**–**d**) Death rates of Canton-S, *Trap1*^+/−^ and *Trap1*^−/−^ flies after Metformin treatment and 3 h of severe hypoxia (<0.3% O_2_) followed by a reperfusion period of 120 h. Data are shown as means ± SEM of 4 independent experiments, including 3 technical replicates per genotype and experiment (total of 240 male flies for each genotype and condition). Welch’s t-test, two-tailed. * *p* < 0.05., *** *p* < 0.001. (**e**) *Trap1* transcript levels after normoxia and hypoxia followed by different reperfusion periods are shown. The graphs present the means ± SEM of 4 independent experiments. Two-way ANOVA followed by Tukey post-hoc. * *p* < 0.05, *** *p* < 0.001. ^#^ *p* < 0.05 indicates significance compared to the corresponding vehicle. ^§^ *p* < 0.05 indicates significance compared to corresponding normoxia.

To assess the effect of Metformin treatment on *Trap1*-deficient flies after hypoxia, we transferred the newly hatched flies on Metformin-supplemented or NaCl vehicle control food up to 5 days and performed 3 h of severe hypoxia followed by 120 h of reperfusion (Figure 4a). We did not observe any differences in the mortality rate of Canton-S between the treated and the vehicle control group. However, *Trap1*-deficient flies showed a significant decrease in post-hypoxic mortality after Metformin treatment (*p* = 0.0002). *Trap1*-deficient flies of the vehicle group reached a mortality rate of 47.18% (*Trap1*^−/−^) and 38.18% (*Trap1^+/^*^−^) during the first 5 days after hypoxia. When treated with Metformin, there was a reduction in mortality of 28.57% and 70.53% to 33.75% in *Trap1*^−/−^ and 11.25% in *Trap1*^+/−^ (Figure 4b–d).

To investigate the impact of Metformin treatment on the expression levels of *Trap1* we subjected Canton-S and *Trap1*^+/−^ flies to 3 h severe hypoxia with a reperfusion period of up to 120 h and assessed the *Trap1* mRNA expression levels. We observed no differences in the mRNA levels under normoxic conditions. However, after hypoxia, both genotypes showed elevated *Trap1* mRNA levels. In Canton-S flies expression levels were significantly increased in the very early reperfusion phase (*p* < 0.0001), whereas the upregulation in *Trap1*^+/−^ flies could be observed up to 3 h after hypoxia (*p* = 0.0174).

Metformin treatment led to a delayed elevation of *Trap1* mRNA levels. Expression levels peaked after 3 h in Canton-S (*p* = 0.0007) and after 6 h in *Trap1*^+/−^ flies (*p* = 0.0003) and returned to normoxic levels after 24 h (Figure 4e).

In conclusion, Metformin rescues the negative effects of *Trap1* deficiency and leads to a later upregulation of *Trap1* in Canton-S and *Trap1*^+/−^ flies.

### 2.5. ROS Production and mRNAs of Antioxidant Proteins Sod, Hsp70 and Catalase Are Upregulated in Trap1-Deficient Flies after Hypoxia, Metformin Attenuates This Upregulation in the Early Reperfusion Period

**Figure 5:**
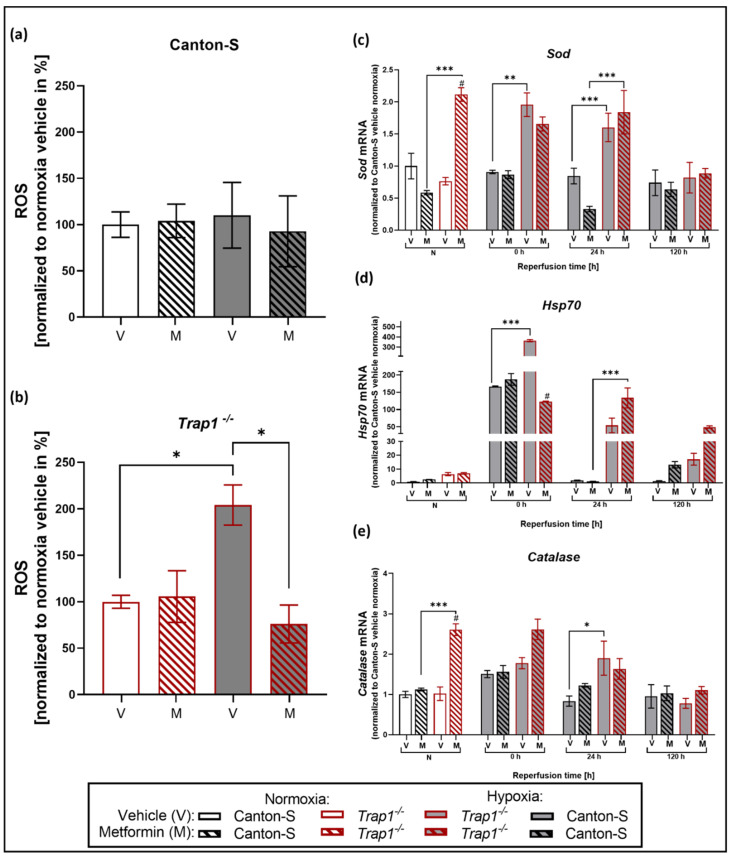
ROS production and post-hypoxic mRNA levels of the antioxidant proteins SOD, HSP70, and Catalase in Metformin or vehicle-treated Canton-S and *Trap1*^−/−^ *D. melanogaster* after 3 h of hypoxia followed by a reperfusion period of up to 120 h. (**a,b**) ROS production in Canton-S and *Trap1*-deficient flies after 24 h of reperfusion normalized to the corresponding normoxia vehicle control. (**c**–**e**) Transcript levels of *Sod, Hsp70*, *and Catalase* after normoxia and hypoxia followed by different reperfusion periods. V stands for the vehicle (NaCl) and M stands for Metformin. Two-way ANOVA followed by Tukey post-hoc. * *p* < 0.05., ** *p* < 0.01, *** *p* < 0.001, ^#^ *p* < 0.05 indicates significance compared to corresponding vehicle.

Assessing the impact of Metformin treatment on the ROS production after hypoxia, we decided to only measure ROS after 24 h of reperfusion, as we expected the highest effect at that reperfusion time in line with the previously described ROS data (Figure 3). We found that Metformin counteracted the previously described significant post-hypoxic elevation of ROS levels in *Trap1*^−/−^ flies (*p* = 0.01) and led to a significant decrease of ROS in *Trap1*^−/−^ flies after hypoxia (*p* = 0.0125) (Figure 5a,b).

The enzymes Superoxide dismutase (SOD), Heat shock protein 70 (HSP70), and Catalase are known to play a role in the antioxidant defense system, which is assumed to influence oxidative stress. To further investigate oxidative stress, we measured the mRNA levels of *Sod*, *Hsp70,* and *Catalase* in the heads of flies after 3 h of severe hypoxia followed by up to 120 h reperfusion. Under normoxic conditions, no differences could be observed between the different genotypes in the vehicle group. After hypoxia, however, *Trap1*^−/−^ flies showed a significant increase in *Sod* (*p* = 0.0004) and *Hsp70* (*p* < 0.0001) expression levels during the first 24 h of reperfusion compared to normoxia, peaking directly after hypoxia at 0 h of reperfusion, whereas Canton-S flies only displayed a significant upregulation of *Hsp70* directly after hypoxia (*p* < 0.0001), which normalized throughout the reperfusion period. Moreover, the mRNA levels of *Sod*, *Hsp70,* and *Catalase* were significantly higher in *Trap1*-deficient flies at nearly all reperfusion times compared to Canton-S. Metformin treatment significantly elevated *Sod* and *Catalase* expression levels in *Trap1*^−/−^ flies under normoxic conditions (*p* < 0.0001). However, Metformin significantly reduced post-hypoxic *Hsp70* (*p* < 0.0001) in *Trap1*^−/−^ flies in the early reperfusion phase (Figure 5c–e). Our results suggest that Metformin rescues the deleterious effects of *Trap1* deficiency by reducing ROS production and displays lower levels of antioxidant proteins. 

### 2.6. Trap1 Deficiency Enhances the UPR Activation after Hypoxia and Metformin Reduces the Activation of the PERK Branch of the UPR

**Figure 6:**
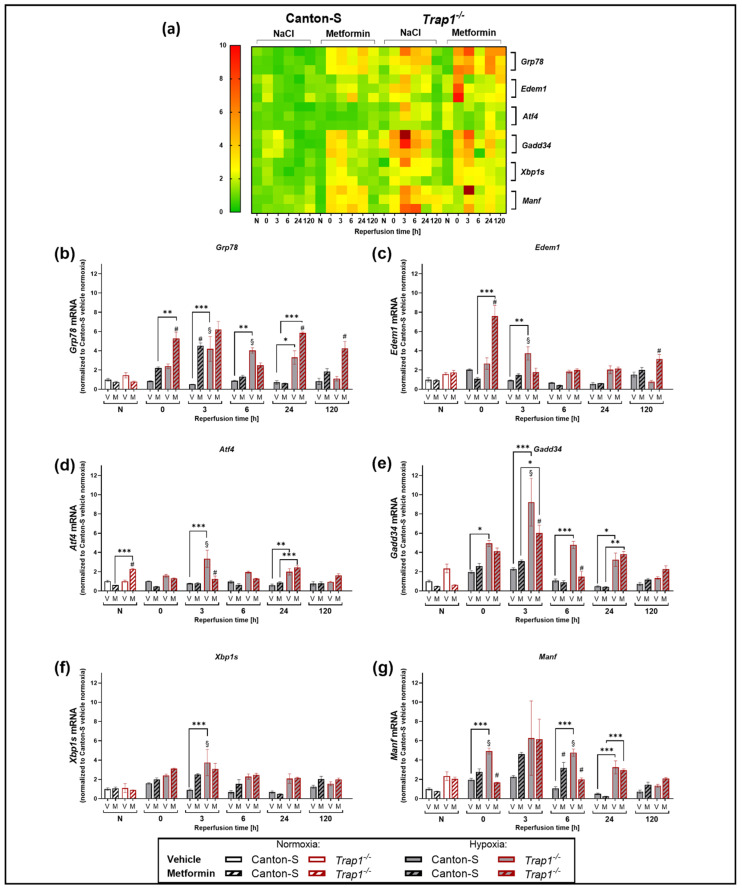
Post-hypoxic mRNA levels of proteins involved in the UPR pathway in Canton-S and *Trap1*^−/−^ *D. melanogaster* after exposure to 3 h of hypoxia. (**a**) Heatmap displaying the mRNA levels of *Grp78, Edem1, Atf4, Gadd34, Xbp1s,* and *Manf* in Canton-S and *Trap1*^−/−^ at different post-hypoxic reperfusion timepoints (0, 3, 6, 24, and 120 h after hypoxia) as a summary of the results of (**b**–**g**). Target genes are presented as ratios to the constitutive gene *eEF1α1* and normalized to the vehicle Canton-S normoxia. (**b**–**e**) Graphs showing the mRNA expression levels of UPR markers. Target genes are presented as ratios to the constitutive gene *eEF1α*1 and normalized to the vehicle Canton-S normoxia. V: vehicle (NaCl) and M: Metformin. Statistics: Two-way ANOVA followed by Tukey post-hoc. * *p* < 0.05., ** *p* < 0.01, *** *p* < 0.001. ^#^ *p* < 0.05 indicates significance compared to the corresponding vehicle. ^§^ *p* < 0.05 indicates significance compared to corresponding normoxia.

To analyze the impact of TRAP1 on the UPR response after hypoxia, we compared the post-hypoxic expression levels of *Grp78, Edem1, Atf4, Gadd34, Xbp1s,* and *Manf* in Canton-S and *Trap1*^−/−^ flies (Figure 6a).Under normoxic conditions, no differences in the expression levels of the UPR markers could be observed between Canton-S and *Trap1*^−/−^ flies. However, hypoxia caused a bell-shaped increase of all UPR markers in the time course of reperfusion, peaking after 3 h of reperfusion. Whereas there was only a mild increase (about a 2-fold) observed in Canton-S flies, UPR markers were strongly increased (2–8-fold) in *Trap1*-deficient flies (Figure 6b–g). Assessing the influence of Metformin treatment on the UPR response, we observed that normoxic conditions displayed no differences between vehicle and treatment group. In Canton-S flies the treatment with Metformin led to increased expression levels of *Grp78* (*p* < 0.0001) (Figure 6b) and *Manf* (*p* < 0.0001) (Figure 6g) after hypoxia. *Atf4, Gadd34*, *Edem1,* and *Xbp1s* showed no significant difference (Figure 6c–f). In *Trap1*^−/−^ flies the expression levels of *Grp78* (*p* = 0.0044) and *Edem1* (*p* < 0.0001) were increased under Metformin treatment after hypoxia compared to the vehicle control (Figure 6b,c). *Atf4* (*p* < 0.0001), *Gadd34* (*p* = 0.0255) and *Manf* (*p* < 0.0001) were decreased following Metformin application compared to the vehicle control (Figure 6d,e,g). *Xbp1s* showed no difference in expression levels between the treatment and vehicle group (Figure 6f).

In conclusion, *Trap1* deficiency seems to affect all three arms of the post-hypoxic UPR. Whilst Metformin administration upregulated mainly downstream of both the ATF6 and IRE1α arms of the UPR in Canton-S after hypoxia, it appeared to upregulate the ATF6 arm while downregulating the IRE1a and PERK arms after *Trap1* deficiency.

## 3. Discussion

In this study, we analyzed the impact of the mitochondrial chaperone TRAP1 on post-hypoxic mitochondrial/ER stress and the activation of the UPR in *D. melanogaster.* In addition, we sought to analyze whether the frequently reported cytoprotective effect of the anti-diabetic drug Metformin is mediated through TRAP1 and the UPR. We demonstrated that both heterozygous (*Trap1^+/^*^−^) and homozygous (*Trap1*^−/−^) *Trap1*-deficient flies were more sensitive to severe hypoxia than the corresponding Canton-S controls, displaying higher mortality rates and increased impairments in negative geotaxis. In addition, we detected a reduction of the median overall survival in *Trap1*-deficient flies in comparison to Canton-S under normoxic and hypoxic conditions. The latter is in line with Costa et al., who also observed a reduced lifespan of *Trap1*-deficient flies and enhanced sensitivity to heat stress, the pesticide paraquat, and the mitochondrial poisons rotenone and antimycin [46]. Under normoxic conditions, the lifespan of heterozygous and homozygous *Trap1*-deficient flies did not differ significantly (Figure 2c). However, upon hypoxia *Trap1*^−/−^ flies exhibited a significantly reduced lifespan compared to *Trap1*^+/−^ flies indicating that the protective role of TRAP1 appears to unfold after hypoxic stress. The same was true in the post-hypoxic assessment of the negative geotaxis. Here, both heterozygous and homozygous *Trap1*-deficient flies displayed similar reduced climbing abilities in the acute phase of reperfusion (Figure 2d). The climbing ability of the *Trap1*-deficient flies recovered to the level of Canton-S later in the reperfusion period. This recovery observed in the *Trap1*-deficient flies may be explained as the result of a selection bias. Note that only viable animals have been considered for the measurement of negative geotaxis. Since the mortality rates of the heterozygous and homozygous *Trap1*-knockout flies were substantially higher than Canton-S, we cannot exclude the possibility that the surviving flies of these genotypes have a higher resistance to hypoxia.

Under normoxic conditions, the homozygous *Trap1*-deficient and Canton-S flies had comparable levels of climbing ability, while the heterozygous *Trap1*-deficiency flies appeared to have a decreased climbing ability. This can be explained by the fact that the heterozygous flies carry a balancer chromosome that can affect the fitness of these flies. As Miller et al., have pointed out, balancers that are kept in stock for an extended period of time may exhibit structural changes that can affect behavioral trials [47].

*Trap1*-deficient flies also presented enhanced ROS production and metabolic activity, higher mRNA levels of antioxidant proteins, and an activation of the UPR compared to the corresponding controls (Canton-S) (Figure 3e–h).

However, it should be noted, that the CTB assay used to assess the metabolic activity only allows an approximate impression of the metabolic state, as it requires intact mitochondria to allow oxidation of resazurin to resorufin. In future studies, metabolic activity should be further investigated by additional assessment of NADPH/FADH_2_ and ATP levels.

Mitochondrial damage and ER stress have been reported to play a pivotal role in the pathophysiology of stroke [48,49]. Previous in vitro experiments in astrocytes revealed that TRAP1 overexpression protected these cells from glucose deprivation (GD) by decreasing ROS levels. In addition, TRAP1 overexpression also preserved ATP levels and the mitochondrial membrane potential during GD [50]. Moreover, overexpression of TRAP1 in rats decreased infarct volumes after experimental stroke and improved their post-ischemic neurobehavioral score. Furthermore, oxidative stress and mitochondrial damage were found reduced after TRAP1 overexpression compared to the corresponding controls [50]. However, the involvement of TRAP1 in ER stress and the subsequent activation of the UPR remains elusive.

The expression of *Trap1* has been found upregulated in various human malignancies, including nasopharyngeal, breast, prostate, and non-small cell lung cancer leading to reduced apoptosis [51,52,53,54]. This reduction of apoptosis is assumed to be caused by the inhibition of the pro-apoptotic CypD-dependent MPTP opening leading to a blockade of the mitochondria-mediated intrinsic apoptotic cascade [55,56]. In addition, TRAP1 is reported to convey its anti-apoptotic properties through an abrogation of ROS production as demonstrated by Zhang and colleagues in a myocardial ischemia model and through its ability to reduce ER stress [55,56].

While under normoxic conditions no obvious differences in the activation of all three arms of the UPR were detected between the genotypes, hypoxia resulted in a relevant increase in the UPR in *Trap1*-deficient flies compared with Canton-S flies. This again points to the crucial role of TRAP1 especially in stress conditions and suggests an important role for TRAP1 in post-hypoxic UPR activation. We assume that elevated ROS levels in *Trap1*-deficient flies might be responsible for the increased ER stress and the subsequent activation of the UPR after hypoxia. This is supported by previous studies demonstrating that elevated ROS production activates the UPR and *Trap1* deficiency leads to higher ROS production [57]. Furthermore, we could also observe that the highly activated detrimental PERK arm of the UPR in *Trap1*-deficient flies was counteracted by Metformin. In line with the assessment of post-hypoxic mortality rates, Metformin did not affect the activation of the PERK arm in Canton-S flies.

Takemoto and colleagues revealed that knockdown of TRAP1 in SH-SY5Y neuronal cells upregulated the expression of glucose-regulated protein (GRP78/BiP) accompanied by a downregulation of CCAAT-enhancer-binding protein homologous protein (CHOP) [25]. Moreover, this study also reported that TRAP1-knockdown cells activated caspase-4 which is observed upregulated upon ER stress and is assumed to regulate the activation of the UPR [25,26]. Interestingly, TRAP1 knockdown cells in this study exhibited downregulation of CHOP, which has been implicated as a pro-apoptotic marker. GRP78/BiP acted similarly in both studies after TRAP1 knockdown or, as in our case, after TRAP1 knockout. However, Takemoto et al., described a reduction of CHOP only after 24 h after pharmacological ER stress using tunicamycin and thapsigargin, whereas GRP78/BiP was upregulated throughout the 24 h. The authors stated that the detrimental effect caused by the absence of TRAP1 might just be postponed by the upregulation of BiP. It is known that the binding of GRP78/BiP to the three UPR sensors respectively abrogates their activation, while CHOP as a downstream of the PERK arm of the UPR may induce cell death during sustained ER stress. Matassa and colleagues observed a decreased activation of PERK followed by reduced phosphorylation of eukaryotic initiation factor-2α (eIF2α) in TRAP1 knockdown cells. Phosphorylated eIF2α enhances the synthesis of selective stress-responsive proteins such as the transcription factor ATF4 and its downstream effector GRP78, leading to protection against ER stress and oxidative damage. Our current study did not assess *Chop*, instead, we measured *Gadd34* and *Atf4* of the PERK-branch of the UPR. Following hypoxia, we observed a bell-shaped regulation curve of the above-mentioned markers of the PERK arm, which peaked after 3 h of reperfusion. However, the *Trap1*-deficient flies presented a higher level of these markers than the wildtype control flies throughout the entire observation phase (Figure 6)

A previous study has reported that a homozygous TRAP1 loss-of-function mutation in fibroblasts derived from one patient was rescued by the antidiabetic drug Metformin [34]. Besides several positive studies on the cytoprotective capacity of Metformin after stroke, there are also conflicting reports [39,58,59]. In fact, administration of Metformin directly after stroke exacerbated brain damage while chronic prophylactic medication with Metformin appeared to be protective improving neurological outcomes in rats [58].

Metformin decreased mortality rates in *Trap1*-deficient flies. However, there was no beneficial effect on the Canton-S flies (Figure 4b). Furthermore, Metformin treatment in *Trap1*-deficient flies decreased ROS production and lowered post-hypoxic mRNA levels of antioxidant proteins such as HSP70 and Catalase suggesting an anti-oxidative property of Metformin [60,61]. In line with Fitzgerald et al., our data provide evidence that Metformin rescues the *Trap1* deficiency [46]. Under normoxic conditions, *Trap1* mRNA of Canton-S flies and *Trap1* heterozygous flies was not upregulated. However, after hypoxia *Trap1* transcripts were found enhanced in Canton-S and *Trap1* heterozygous flies. This indicates that *Trap1* is upregulated in response to hypoxic stress in agreement with the suggested protective role of *Trap1*. After Metformin treatment, however, we observed significantly reduced *Trap1* mRNA levels in Canton-S and *Trap1*-deficient flies compared to the vehicle group. In association with the evidence that ROS was reduced after Metformin treatment in *Trap1*-deficient flies, we assume that Metformin counteracts oxidative stress through an alternative non-TRAP1 dependent pathway. If this pathway is as potent as the TRAP1 pathway, it might also explain why the ROS production shows no significant difference with or without Metformin treatment in Canton-S flies. Our results suggest that TRAP1 plays a crucial role in mitochondrial homeostasis impeding oxidative stress and resolving ER stress after hypoxia. A possible explanation for the lack of neuroprotection in Canton-S flies might be that the induced hypoxic stress and resulting mortality rates were too low to be affected by Metformin. In addition, the administration of Metformin might have been too short in regards to the lifespan of the Canton-S flies. To ensure the consistency of our experiments and the comparability of our results we decided to use flies that were treated with Metformin for 1–5 days. As a result of different treatment periods, a high variability could occur in the outcome and influence the statistics. Further studies are needed to investigate the effects of longer Metformin treatment.

## 4. Materials and Methods

### 4.1. Drosophila melanogaster

*D. melanogaster* wild-types Canton-S (#64349) were obtained from the Bloomington *Drosophila* Stock Centre (Bloomington, IN, USA). *Trap1* mutant flies (w*; Trap1Δ4/CyO #58767) were generated by an imprecise excision of the *p*-element and kindly provided by Miguel Martins [46]. All flies were raised and maintained in plastic vials containing standard cornmeal food at 23 °C under a 12 h/12 h light/dark cycle. Every five days, flies were transferred to new vials with fresh food.

### 4.2. Metformin Treatment

Metformin (STEMCELL Technologies #73254, Cologne, Germany) was added to standard cornmeal food at 55 °C to obtain a final concentration of 10 mM. Flies were put on the Metformin for 1–5 days prior to hypoxia and for the reperfusion period of 5 days.

### 4.3. Hypoxia Chamber

As previously described [45] hypoxia was induced with nitrogen (N_2_) in a self-constructed hypoxia chamber, consisting of a heated water chamber, gas-flow regulator, humidifier, and an airtight acrylic glass compartment with a stainless-steel bottom plate and lid (Figure 1a). The chamber enables control and monitoring of oxygen levels (Greisinger GOX 100 T O_2_ – Sensor, Greisinger, Regenstauf, Germany), humidity, temperature (Habor Thermo-Hygrometer, Habor, Deggenhausertal, Germany), and pressure (Fluke 700RG06 100 PSIG pressure gauge, Fluke, Glottertal, Germany).

### 4.4. Mortality Rate

To study the impact of hypoxia on survival, male Canton-S, *Trap1*^+/−^ and *Trap1*^−/−^ flies from age 1 to 5 days were subjected to 1 to 6 h of severe hypoxia (<0.3% O_2_). The mortality rate was assessed daily for the following 5 days under normoxic conditions. Temperature, pressure, and humidity inside the chamber were recorded regularly during this time. Due to slight differences between sexes, the following experiments were conducted with male flies only. Each experiment was repeated 4 times for the examined hypoxia period. Per experiment and genotype 3 technical replicates were used. We utilized a total of 240 male flies for each condition and genotype. We subjected all flies only once to a hypoxia period. After the establishment of the half-lethal hypoxia duration, the following experiments were conducted utilizing 3 h of severe hypoxia (<0.3% O_2_).

### 4.5. Drosophila Activity Monitoring Assay (DAM)

The activity of each fly was assessed as previously reported [45]. After hypoxia, each fly was transferred into the *Drosophila Activity Monitoring* system (Model DAM2, Trikinetics Inc., Waltham, MA, USA). Each fly was placed individually in a polycarbonate vial containing food (2% agar and 4% sucrose). The activity was monitored by recording the light beam interruptions, e.g., caused by the fly passing through, in the center of each. The flies were kept in the DAM system for a total of 5 days, during which the number of interruptions of the light beams was recorded every hour. The experiment was conducted 3 times in total, including 60 flies for each genotype and treatment.

### 4.6. Negative Geotaxis Assay

The negative geotaxis assay was conducted as described previously [62] with slight modifications. The climbing ability was assessed 3, 6, 24, 48, 72, 96, and 120 h after hypoxia and each vial contained a group of 20 male flies. 

### 4.7. Semiquantitative PCR and RT-qPCR

Gene expression analyses were performed as previously reported [38] with tissue from fly heads. In brief: snap-frozen fly heads were homogenized in PeqGold (PeqLab #30–2010, Erlangen, Germany) and total RNA was prepared by phenol-chloroform extraction as previously described [56]. Complementary DNA was synthesized using the iScriptTM cDNA Synthesis Kit (BioRad Laboratories, Hercules, CA, USA) and random hexanucleotide primers (Invitrogen, Germany) using 1 µg of total RNA according to the manufacturer’s protocol. RNase free H2O (Merck, 64293, Darmstadt, Germany) served as no template control (NTC). Semiquantitative PCR was used for genotyping of flies after cDNA synthesis with the following configurations: 3 min at 95 °C, 30 cycles of 30 s at 95 °C, 30 s at 60 °C, and 30 s at 72 °C, followed by 72 °C for 3 min, and a 20 °C hold (T Professional Basic, Biometra, Göttingen, Germany). PCR was performed using the Trap1 primers (fwd: TTCCCGCAGAACAGCAGAAT, rev: TCGTTGCGCTTCTTCAGACT) and Elongation factor 1-alpha 1 (eEF1α1) primers (fwd: ATCCGTCGCGCTTAGACTT, rev: CCCTTTCCCATCTCCTGGGC) as a housekeeper gene. The PCR products were loaded into the sample wells of a 2% agarose gel, supplemented with a 1:25.000 dilution of Midori Green (Biozym Scientific #617004, Hessisch Oldendorf, Germany) and gel electrophoresis was performed at 120 V for 25 minutes followed by visualization of the PCR product using UV transillumination (UVSolo, Biometra, Göttingen, Germany). Quantitative gene expression levels were assessed with tissue from fly heads collected after 3 h of hypoxia or normoxia at different reperfusion timepoints. RT-qPCR analysis was performed using the MyIQ RT-qPCR detection system (Bio-Rad, München, Germany). The target genes (see below) and housekeeping gene (*eEF1α1*) were measured as cycle threshold (Ct values) and relative quantification was calculated by the ΔΔCt method using the qbase+ software (Biogazelle, Gent, Belgium). Data are expressed as the relative amount of the housekeeper gene *eEF1α1*. The following forward (fwd) and reverse (rev) primers were used (5’→3’): *eEF1α1* (fwd: ATCCGTCGCCGCTTAGACTT, rev: CCCTTTCCCATCTCCTGGGC), *Grp78* (fwd: TCTTGTACACACCAACGCAGG, rev: CAAGGAGCTGGGCACAGTGA), *Edem1* (fwd: GAAGCAGTATTCCAAGGCAAGA, rev: GGCGCAGGTAACCATCGTAG), *Atf4* (fwd: AGGCCATAGTACCCGCAAAC, rev: CCGCCTGTTTGTAAGCATCG), *Gadd34* (fwd: CGAGCAATATCGGTTCGGG, rev: TGATGCACCTTGTTTGGCTTC), *Xbp1s* (fwd: CTCGAGTTCGGGATACGCAT, rev: CCAGGTTAGATGGTCCAGGC), *Manf* (fwd: AGATCGAAACGGCCTTCAAAA, rev: GTGGCGGATTCTTCCAGACC), *Sod* (fwd: GGAGTCGGTGATGTTGACCT, rev: GTTCGGTGACAACACCAATG), *Hsp70* (GCTGACGTTCAGGATTCCAT, rev: CGGAGTCTCCATTCAGGTGT), *Catalase* (fwd: ACCAGGGCATCAAGAATCTG, rev: AACTTCTTGGCCTGCTCGTA), *Trap1* (fwd: TTCCCGCAGAACAGCAGAAT, rev: TCGTTGCGCTTCTTCAGACT). 

### 4.8. Metabolic Activity Assay

The CellTiter-Blue^®^ Cell Viability assay (Promega, Madison, WI, USA) was used to assess the metabolic activity after hypoxia. This test was performed as previously described [63]. Procedure in brief: 40 fly heads were homogenized using the Speedmill P12 (Analytik Jena AG, Jena, Germany) in 1 mL of 20 mM Tris buffer, pH 7.0, and centrifuged at 1600 g for 10 minutes at 4 °C. The supernatant was incubated with 0.2 mg/mL resazurin for 4 h. The fluorescence of the converted resazurin was measured at an excitation wavelength of 573 nm and an emission of 584 nm. The metabolic activity of Canton-S was evaluated 0, 6, and 24 h after hypoxia.

### 4.9. ROS Assay

To determine the ROS level after hypoxia, fly head lysates were incubated in 2′,7′-dichlorodihydrofluorescein diacetate (DCFH-DA) as previously described [64]. The fly head lysates were incubated with 5 mM DCFH-DA for 30 min and centrifuged at 400 g for 5 minutes at 4 °C. The pellet was washed once and resuspended in Tris buffer. Fluorescence of DCFH-DA was measured at an excitation wavelength of 488 nm and emission of 525 nm. The ROS production of Canton-S and *Trap1*-deficient flies were evaluated at 0, 24, and 120 h after hypoxia.

### 4.10. Statistics

Data analysis and visualization were performed using GraphPad Prism (version 8.4.3, San Diego, CA, USA). Data of each experiment comprises three to four independent experiments with three technical replicates. Residuals were analyzed for normal distribution using the Shapiro–Wilk and D’Agostino–Pearson omnibus normality test. Variance homogeneity was tested using the Bartlett test or the Spearman’s rank correlation test for heteroscedasticity. For the identification of outliers, a ROUT test was utilized. In case the normality or homogeneity tests were significant, instead of one-way or two-way ANOVA, non-parametric tests were applied. Data are given as arithmetic means ± SEM. The level of significance was set at *p* < 0.05. Asterisks indicating significance between group differences, “#” compares hypoxia vs. the corresponding vehicle (Figure 4e and Figure 6b–g) and “§” compares hypoxia to the corresponding normoxia (Figure 3a–h, Figure 4e and Figure 6b–g). The individual data of each experiment are shown in the legends.

## 5. Conclusions

We provide evidence that TRAP1 is a crucial regulator of mitochondrial and ER stress and counteracts the pro-apoptotic PERK-arm of the UPR. Metformin rescues *Trap1* deficiency but has no beneficial effect on Canton-S flies after hypoxia (graphical abstract). Further studies with different Metformin concentrations and animal models are needed to fully understand the effects conveyed by Metformin.
